# Reliability of left ventricular hemodynamic forces derived from feature-tracking cardiac magnetic resonance

**DOI:** 10.1371/journal.pone.0306481

**Published:** 2024-07-25

**Authors:** Temirlan Ismailov, Zaukiya Khamitova, Dinara Jumadilova, Nail Khissamutdinov, Bauyrzhan Toktarbay, Nurmakhan Zholshybek, Yeltay Rakhmanov, Alessandro Salustri

**Affiliations:** 1 Nazarbayev University School of Medicine, Astana, Kazakhstan; 2 National Research Cardiac Surgery Center, Astana, Kazakhstan; Okayama University: Okayama Daigaku, JAPAN

## Abstract

**Background:**

Hemodynamic forces (HDF) analysis has been proposed as a method to quantify intraventricular pressure gradients, however data on its reliability are still scanty. Thus, the aim of this study is to assess the reliability of HDF parameters derived from cardiac magnetic resonance (CMR).

**Methods:**

CMR studies of 25 athletes were analysed by two independent observers and then re-analysed by the same observer one week apart. Intraclass Correlation Coefficient (ICC [95% CI]) and Bland-Altman plots were used to assess association, agreement, and bias of the longitudinal (A-B) HDF, transverse (L-S) HDF, and Impulse Angle. The sample size required to detect a relative change in the HDF parameters was also calculated.

**Results:**

In terms of inter-observer variability, there was a good correlation for the A-B and L-S (ICC 0.85 [0.67–0.93] and 0.86 [0.69–0.94]; p<0.001 for both, respectively) and a moderate correlation for the Impulse Angle (ICC 0.73 [0.39–0.87]; p = 0.001). For intra-observer variability, A-B and L-S showed excellent correlation (ICC 0.91 [0.78–0.93] and 0.93 [0.83–0.97]; p<0.001 for both, respectively). Impulse Angle presented good correlation (ICC 0.80 [0.56–0.90]; p<0.001). Frame selection and aortic valve area measurements were the most vulnerable step in terms of reliability of the method. Sample size calculation to detect relative changes ranged from n = 1 to detect a 15% relative change in Impulse Angle to n = 171 for the detection of 10% relative change in A-B HDF.

**Conclusions:**

The results of this study showed a low inter- and intra-observer variability of HDF parameters derived from feature-tracking CMR. This provides the fundamental basis for their use both in research and clinical practice, which could eventually lead to the detection of significant changes at follow-up studies.

## Introduction

The analysis of myocardial function has been based so far on the evaluation of left ventricular wall motion (either regional or global) [1] or on the velocity of the blood within the heart [2], which are both surrogates of the intrinsic myocardial contractile properties. In fact, the development of intraventricular pressure gradients is responsible for blood flow within the heart and large vessels [3]. Recently, the quantification of intraventricular pressure gradients using hemodynamic forces (HDF) analysis has been proposed as a new approach for a complete assessment of cardiac hemodynamics and for analysing the strength with which blood propagates within the left ventricle (LV) [4,5].

The importance of new parameters for detecting LV dysfunction is related to the limitations of the current methods, both for systolic as well as diastolic dysfunction. For example, LV ejection fraction (EF) is based on volume ratios (and not on the intrinsic myocardial contractile state) [6], strain evaluates myocardium only (not the dynamic of the blood) and has intrinsic technical limitations [7], and doppler methods for assessing LV diastolic dysfunction are flow-dependent [8]. Thus, it is important to highlight the physiological significance and the potential clinical relevance of HDF analysis, which could lead to the early detection of LV systolic and diastolic dysfunction.

This method has been applied in patients with dilated cardiomyopathy [9] and in patients with heart failure (HF) [10,11], as well as in athletes [12]. Furthermore, it could be ideally applied to follow-up studies in patients with chronic heart disease such as: post-myocardial infarction (MI) or HF with reduced ejection fraction for the detection of subtle changes over time in cardiac function and hemodynamics [13,14]. However, a reliable comparison of studies in each patient at different times requires variability of the measurements as low as possible to differentiate between real changes due to the progression of the disease and fictitious changes due to the intrinsic variability of the measurements. Thus, despite the great potential interest for HDF analysis, its wide acceptance and clinical relevance need solid data on the reliability which are still lacking.

With these concepts in mind, we sought to assess the inter- and intra-rater reliability of the HDF parameters derived from feature tracking CMR and to determine the relative weight in terms of variability of the several steps of HDF analysis. Moreover, we calculated the necessary study sample size to define the number of subjects required for future studies.

## Materials and methods

### Study population

This research is part of an ongoing study that evaluates the effect of intense physical training on HDF in athletes. Briefly, athletes of sport federations trained at the Athletic Center of Nazarbayev University in Astana, Kazakhstan, will undergo CMR at the end of the off-season and the HDF parameters derived from the CMR images will be compared with those obtained after three and six months of intense physical training and with those obtained in a group of patients with longstanding hypertension. The study was approved by the Ethical Committee of Nazarbayev University (NU-IREC #550/20042022).

For the purpose of this study, 25 endurance athletes >18-years old were randomly selected and then analysed for inter-observer variability and intra-observer variability.

### Cardiac MR acquisition

Cine CMR studies were performed at the National Research Cardiac Surgery Center in Astana, Kazakhstan, using a Siemens Magnetom Avanto 1.5 Tesla machine. The CMR scanning protocol was based on the "Cardiovascular Magnetic Resonance Pocket Guide" [15]. The localiser scans were acquired using True Fast Imaging with Steady-State Free Precession (TRUFI), multi 2Ch view, Institute for Personality and Ability Testing (IPAT), and four-chamber (4Ch) view. These were followed by white-blood image acquisitions in long-axis 4Ch-, 2Ch-, and 3Ch-, and short-axis views. For all acquisitions the distance factor was kept at 20% with a slice thickness of 8 mm, an interslice gap of 2 mm, a repetition time (TR) of 34.68ms, and an echo time (TE) of 1.22ms. After the acquisition, all the images were stored in the Picture Archiving and Communication System (PACS) and retrieved for off-line analysis.

### Analysis of CMR images

Images were analysed by two independent observers (DJ, NK) with >6 years of experience in cardiac imaging using a dedicated software (Q Strain version 1.3.0.79; Medis, Leiden, the Netherlands) which allows to calculate HDF by visualising three routine long-axis and one short-axis cine randomly pre-selected images from PACS. The algorithm is based on a mathematical model [16]:

$$F(t)=\rho \int_{S(t)}[x(\frac{\partial v}{\partial t}*n)+v(v*n)]dS$$ (1)

Before starting this study, three joint reading sessions on analysis of clinical routine CMR studies were conducted for standardisation of measurements between the two observers.

**Fig 1:**
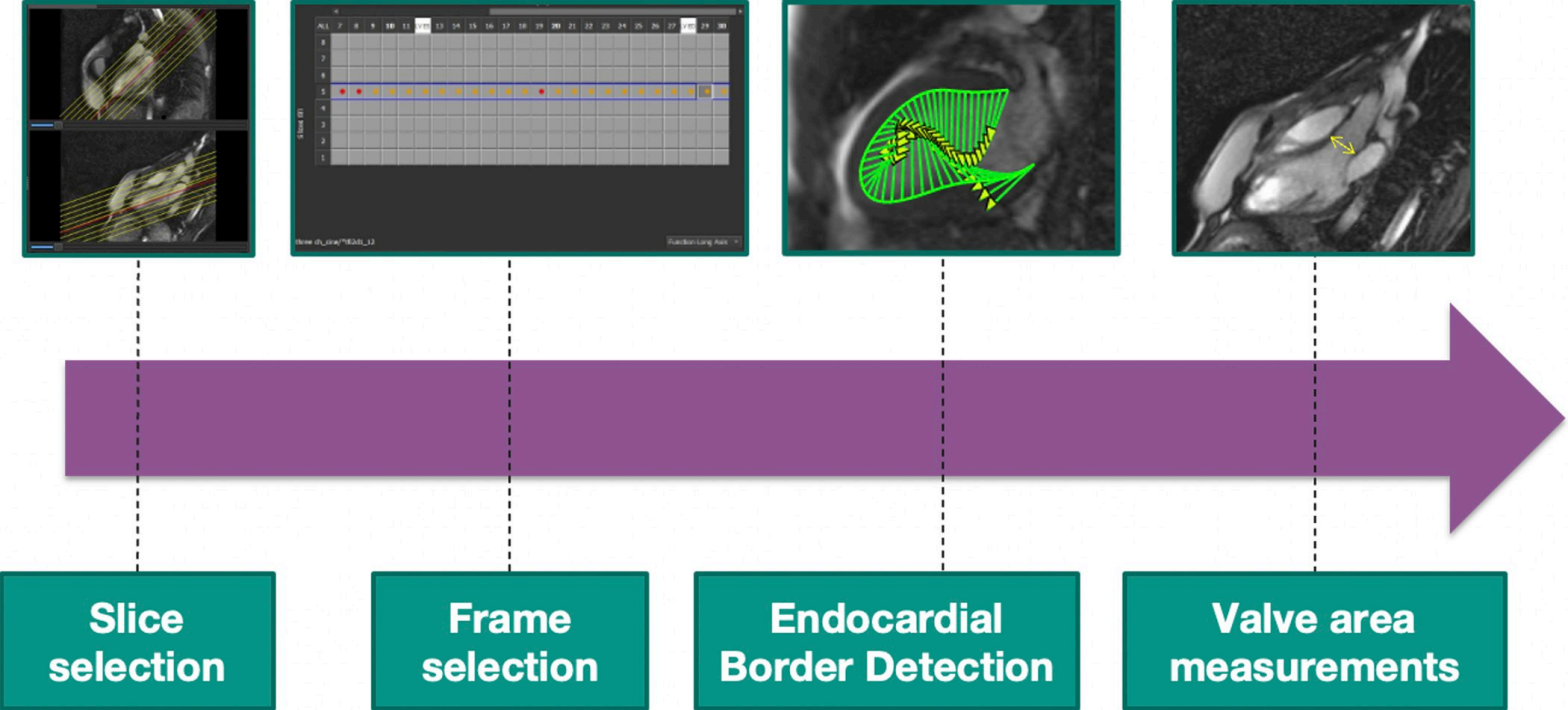
The main steps of HDF analysis. First, the observer selects and then imports the CMR images. Then, the observers scroll the cardiac loop to select the end-systolic (ES) and end-diastolic (ED) frame. At this point, the software automatically performs the endocardial border detection for feature-tracking, however the operator has the freedom to manual adjustment, if needed. Finally, aortic valve (AV) and mitral valve (MV) diameter are measured for valve area assessment.

The main steps of HDF analysis are summarized in **Fig 1**.

To perform a HDF analysis, a 2, 3, and 4Ch cine image is needed. During slice selection, in CMR images with perfect planimetry the observers selected the mid slice from a stack. However, in cases when planimetry was not done accurately during scanning (i.e. for MV—in both 4Ch and 2Ch views—the midpoint of the valve orifice and the cardiac apex; for AV—in 3Ch view—the center of AV, MV, and the cardiac apex), the observers had to search for a compromise when selecting the slice to make it as closest as possible to these criteria.

Based on the selected slice, the observers could observe consecutive cardiac frames on each long-axis view to select the end-systole (ES) and end-diastole (ED) time points, if they were not already automatically detected.

Then, the left ventricular endocardial border was automatically detected by the software in the appropriate cardiac frame. The observers had the freedom to manually change the endocardial borders automatically selected by the program, if needed. In addition to HDF, the endocardial contouring feature allowed the calculation of the Global Longitudinal Strain (GLS) and standard volumetric parameters (such as the LV volumes and EF), which are dependent on endocardial border detection (EBD).

Finally, the calculation of the LV HDF requires measurements of the mitral valve area (MVA) and aortic valve area (AVA). The MVA was calculated from the measurement of the mitral annulus diameter (A = π [d/2]^2^) and the measurements were performed only after the observers selected the appropriate cardiac frame with maximal MV opening. The MVA was calculated from the 4Ch and 2Ch views and the mean of the two values were considered. The AVA was calculated from the aortic annulus diameter at end-systole from the inner-edge to the inner-edge of the vessel in the 3Ch view. For each structure (MV and AV), three measurements of the diameters were performed, and the values were averaged and inserted manually into the software for automatic calculation of the MVA and AVA.

### HDF parameters (Fig 2)

In the normal LV, HDF occurs in the apical-base (A-B) and lateral-septal (L-S) planes, with the A-B direction being the most predominant force. The A-B direction of the forces is depicted as a curve that deflects positively when HDF is directed toward the LV base and negatively when it is directed toward the LV apex. The Newton (N) is the unit of force. Typically, the root mean square, which includes both positive and negative values, is used to express the amplitude characteristics of HDF. The value of HDF is divided by the fluid density and gravity acceleration after being initially normalised to the equivalent LV volume. This makes it possible to describe the acceleration due to gravity as a percentage (dimensionless) and makes it easier to compare subjects. For the purpose of the study, HDF along the entire cardiac cycle have been calculated. Furthermore, the impulse angle designating the dominant force vector during the entire cardiac cycle ranging from 90° (when the force is perfectly along the base-apex direction) to 0° was automatically calculated by the software to express the orientation parameters of HDF [17].

**Fig 2:**
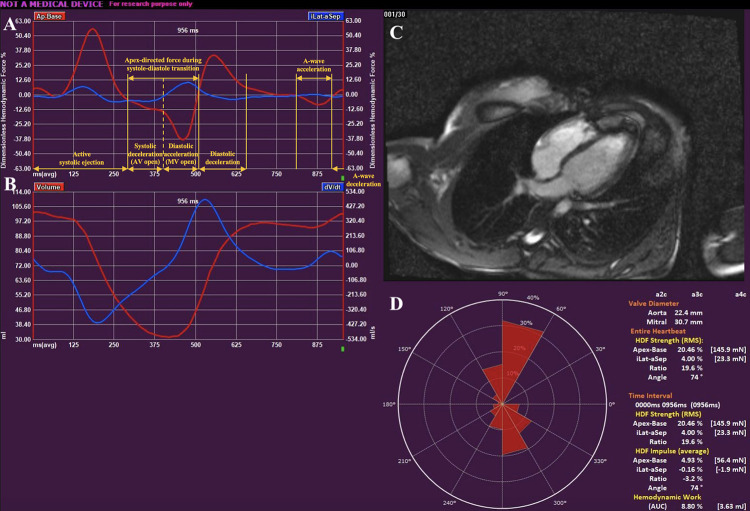
Hemodynamic forces analysis. **(A)** HDF curves (A-B in red; L-S in blue). Several time intervals can be defined from the HDF curve including active systolic ejection, systolic deceleration, diastolic acceleration, diastolic deceleration, atrial thrust, and atrial deceleration; **(B)** LV volume and rate of changes in LV volume curve; **(C)** Cardiac MRI image; **(D)** Polar histogram depicting the distribution and magnitude of LV HDFs. The magnitude (red triangles) and directional distribution (angles) of HDF across the entire heart cycle are both depicted on a polar histogram. The top of the histogram (90 degrees) is used to represent the LV base, while the bottom (270 degrees) indicates the LV apex.

### Statistical methods

For inter- and intra-observer variability, the distribution of the measurements within each group or pair was assumed to be approximately normal. The association, bias, and agreement were assessed and interpreted according to the standard definition [18]. All statistical analyses were carried out with Stata 18.0 software. The study was approved by the Ethical Committee of Nazarbayev University (NU-IREC #550/20042022). Informed consent was obtained from all individual participants included in the study.

- **Association**. *Intraclass correlation coefficient*. The intraclass correlation coefficient (ICC) and the 95% C.I. were used to assess the reliability of consistency of measurements made on the same patients. Two-way mixed effect model, single-rater, absolute agreement ICC was used for quantifying the consistency in single operator’s (intra-rater) measurements over time, and mean-rating (k = 3) ICC for the reliability of measurements made by different operators (inter-rater) [19]. The ICC value ranges between 0 and 1, where values <0.5 indicate poor reliability, values between 0.5 and 0.75 moderate reliability, values between 0.75 and 0.9 good reliability, and values >0.9 excellent reliability. A p value of <0.05 was considered statistically significant.
- **Bias and agreement**. *Bland-Altman plot*. The degree of agreement between different measurements was assessed by constructing the Bland-Altman plot. At this aim, 1) the difference in the pair of measurements were plotted against their averages, 2) the bias (the mean of the differences) were calculated and plotted, and 3) the upper and lower limits of agreement (bias +/- 1.96 x SD of difference) were calculated and plotted. The limits of agreement indicate the range within which most of the differences between operators fall. The interpretation of the Bland-Altman plot depends on the clinical magnitude of the limits and involves considering several aspects: agreement and bias. The level of agreement between the observers is higher the closer the differences that cluster around zero. If the differences deviate from zero on a consistent basis, it indicates the presence of a systematic bias, showing a continuous difference in measurements between the observers.

### Sample size calculation

Study sample size required to detect a relative % change in HDF with power of 90% and significance of 5% was calculated as follows [20]:

$$n=f(\alpha ,P)*\sigma^{2}*\frac{2}{\delta^{2}}$$ (2)

where *n* is the sample size, α the significance level, *P* the study power required and *f* the value of the factor for different values of α and *P* (*f* = 10.5 for α = 0.05 and p = 0.090), with σ the standard deviation of differences in measurements between two studies and δ the desired difference to be detected. Sample size calculation was performed for inter-observer variability values only.

## Results

The general characteristics of the athletes and the basic CMR parameters analysed by the two observers are dysplayed in **Table 1**. The gender was equally distributed, the mean age was 32.9 ± 11.9 years, and the mean length of experience in sport was 12.0 ± 7.6 years.

**Table 1:** Demographic, clinical characteristics, and basic CMR data of 25 athletes.

Age, years (SD)	32.9 (11.9)
Female, n (%)	11 (44.0)
Male, n (%)	14 (56.0)
Weight, kg (SD)	70.8 (16.1)
Height, cm (SD)	170.8 (9.3)
BSA^a^, m^2^ (SD)	1.82 (0.25)
HR^a^, bpm (SD)	64.2 (12.7)
SBP^a^, mmHg (SD)	115.9 (13.7)
DBP^a^, mmHg (SD)	77.0 (10.9)
MAP^a^, mmHg (SD)	90.0 (11.4)
Sport experience, years (SD)	12.0 (7.6)
LVM^a^, g (SD)	97.4 (29.4)
LVMi^a^, n (SD)	50.0 (20.3)
LVESV^a^, mL (SD)	57.8 (20.7)
LVESVi^a^, n (SD)	31.7 (11.2)
LVEDV^a^, mL (SD)	148.4 (39.4)
LVEDVi^a^, n (SD)	81.7 (20.7)
LVEF^a^, % (SD)	61.8 (7.1)

^a^BSA, body surface area; DBP, diastolic blood pressure; HR, heart rate; LVEF, left ventricular ejection fraction; LVEDV, left ventricular end-diastolic volume; LVEDVi, left ventricular end-diastolic volume indexed; LVESV, left ventricular end-systolic volume; LVESVi, left ventricular end-systolic volume indexed; LVM, left ventricular mass; LVMi, left ventricular mass indexed; MAP, mean arterial pressure; SBP, systolic blood pressure.

### HDF—inter-observer variability

The ICC values showed good correlation for A-B HDF (0.85[0.67–0.93]; p<0.001) and L-S HDF (0.86[0.69–0.94]; p<0.001), while that for the Impulse Angle showed moderate correlation (0.73[0.39–0.87]; p = 0.001) (**Table 2**).

**Table 2:** Inter- and intra-observer variability for HDF parameters.

Inter-observer variability Intra-observer variability				
Parameter	ICC (95% C.I.)	p-value	ICC (95% C.I.)	p-value
Hemodynamic forces				
A-B	0.85 (0.67–0.93)	<0.001	0.91 (0.78–0.93)	<0.001
L-S	0.86 (0.69–0.94)	<0.001	0.93 (0.83–0.97)	<0.001
Impulse Angle	0.73 (0.39–0.87)	0.001	0.80 (0.56–0.90)	<0.001

**Fig 3:**
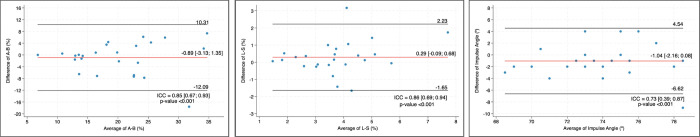
Bland-Altman plots of agreement between the two observers: Longitudinal (A-B) HDF, transverse (L-S) HDF, and the impulse angle.

**Fig 3** shows Bland-Altman plots of agreement between the measurements of the two observers. For all the HDF parameters, minimal bias is present since the middle line of agreement on the y axis is close to zero and 96% of differences are within the limits of agreement.

### HDF—intra-observer variability

While ICC values showed excellent correlation for HDF A-B (0.91[0.78–0.93]; p<0.001) and HDF L-S (0.93[0.83–0.97]; p<0.001), Impulse Angle showed good correlation (0.80[0.56–0.90]; p<0.001) (**Table 2**).

**Fig 4:**
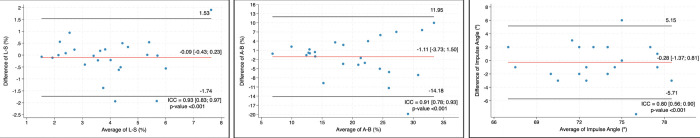
Bland-Altman plots of agreement in repeated measurements by the same observer of the longitudinal (A-B) and transverse (L-S) HDF, and the impulse angle.

The bias trend can also be visualised in **Fig 4** showing Bland-Altman plots of agreement between the same observer measurements. For all the HDF parameters, minimal bias is present since the middle line of agreement on the y axis is close to zero and 92% measurements are within the limits of agreement.

### Reliability of the steps for HDF analysis

- **Slice selection**. In terms of the slice selection, the two observers selected the slices in 2Ch, 3Ch, and 4Ch views with a difference of ≤2 slices in 25/25 (100%) cases, with the same observer showing this degree of variability in 25/25 (100%) cases.
- **Frame selection**. While the ICC for the selections of the two observers for ES frames indicated good correlation (0.85[0.65–0.93]; p<0.001), those for ED frames indicated moderate correlation (0.70[0.56–0.81]; p = 0.004). ICC for measurements of the same observer one week apart showed good correlation for ES (0.82[0.57–0.92]; p<0.001) and ED (0.87[0.72–0.94]; p<0.001).
- **Endocardial border detection (Table 3)**. Since both volumetric data and GLS parameters are dependent on EBD, we calculated the variability of those measurements as an index of the EBD variability. ICC values of inter-observer measurements showed good correlation for GLS (0.89[0.74–0.95]; p<0.001) and LV ejection fraction (EF) (0.83[0.61–0.92]; p<0.001). ICC values of intra-observer variability measurements showed excellent correlation for GLS (0.93[0.83–0.96]; p<0.001) and LVEF (0.98[0.94–0.99]; p<0.001). All volumetric parameters showed excellent inter- and intra-observer variability (ICC >0.9; p<0.001 for all the parameters).

**Table 3:** Inter- and intra-observer variability for volumetric and feature tracking analysis parameters.

Inter-observer variability Intra-observer variability				
Parameter	ICC (95% CI)	p-value	ICC (95% CI)	p-value
Feature tracking analysis				
GLS-endo	0.89 (0.74–0.95)	<0.001	0.93 (0.83–0.96)	<0.001
Volumetric				
LVEF^a^	0.83 (0.61–0.92)	<0.001	0.98 (0.94–0.99)	<0.001
LVESV^a^	0.95 (0.89–0.98)	<0.001	0.98 (0.96–0.99)	<0.001
LVESVi^a^	0.93 (0.85–0.97)	<0.001	0.98 (0.96–0.99)	<0.001
LVEDV^a^	0.98 (0.97–0.99)	<0.001	0.98 (0.97–0.99)	<0.001
LVEDVi^a^	0.98 (0.97–0.99)	<0.001	0.98 (0.96–0.99)	<0.001
SV^a^	0.96 (0.91–0.98)	<0.001	0.98 (0.96–0.99)	<0.001
SVi^a^	0.95 (0.88–0.98)	<0.001	0.98 (0.96–0.99)	<0.001

^a^LVEDV: Left Ventricular End-Diastolic Volume; LVEDVi: Left Ventricular End-Diastolic Volume indexed; LVESV: Left Ventricular End-Systolic Volume; LVESVi: Left Ventricular End-Systolic Volume indexed; SV: Stroke Volume; SVi: Stroke Volume Indexed.

**d. Valve diameter measurement**. While the ICC for the AVA showed good correlation between different observer measurements (0.78[0.51–0.90]) (p<0.001), that for the MVA presented an excellent correlation (0.92[0.67–0.98]) (p<0.001). Similarly, for the same observer one week apart, ICC values for AVA and MVA presented a good and excellent correlation (0.86[0.77–0.95]; p<0.001) and (0.90[0.78–0.96]; p = 0.004), respectively.

### Sample size calculation

**Fig 5:**
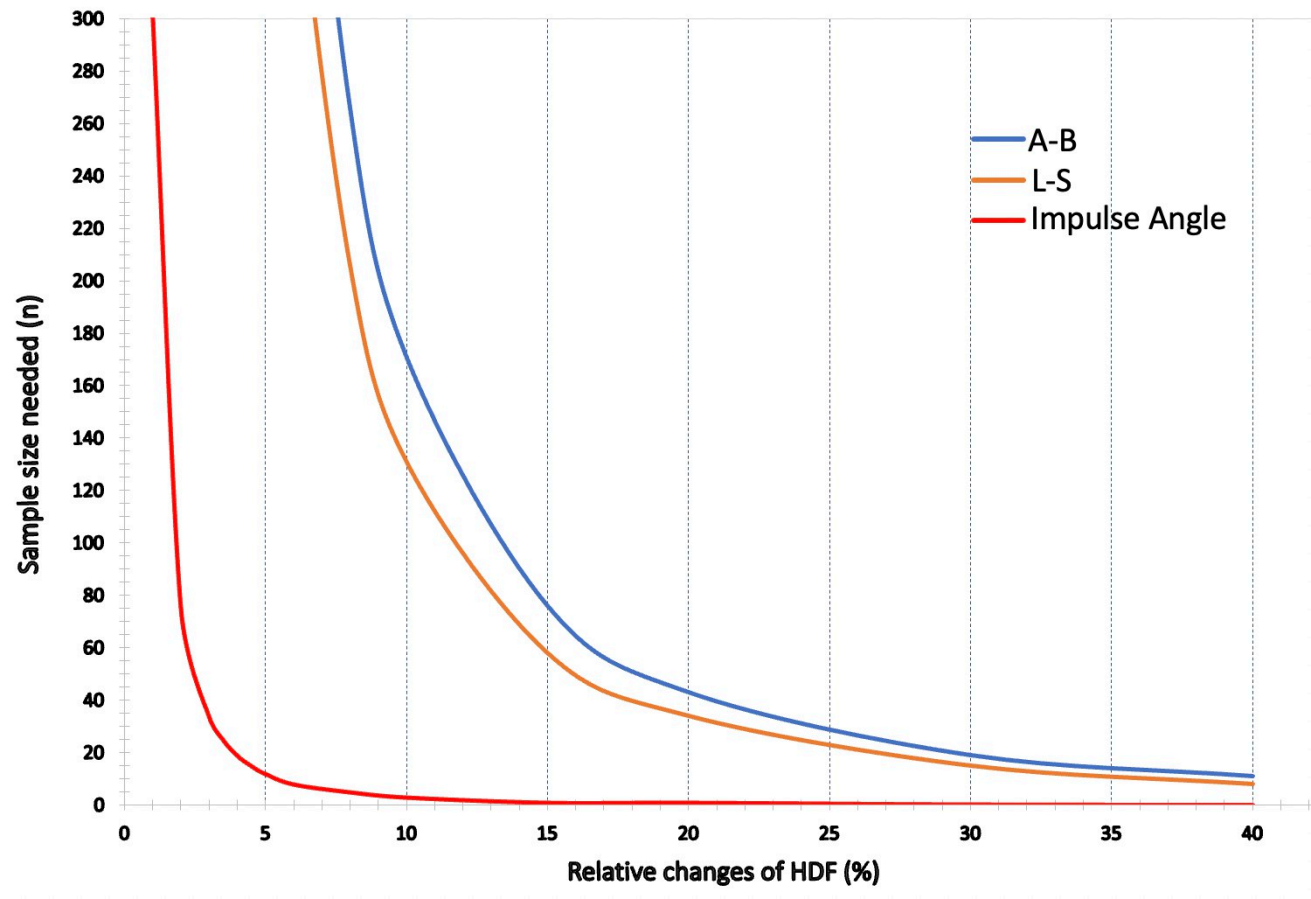
Graphical representation of the sample size calculation for A-B (blue), L-S (yellow), and Impulse Angle (red) to detect % relative changes of HDF with 90% power and α error of 0.05.

The change in reproducibility has an impact on the sample size required to detect significant differences in the HDF parameters. **Fig 5** displays the required sample sizes for each parameter, including longitudinal and transversal hemodynamic forces, and impulse angle. For example, demonstrating a relative 10% change in impulse angle would necessitate only 3 patients. In contrast, 171 patients are required to detect a 10% change in the A-B. In the case of L-S, the sample size needed to detect a 10% relative change is 131.

## Discussion

In this study, we sought to analyse the reliability of HDF parameters as derived from feature-tracking CMR imaging in athletes. At this aim, we applied the ICC to evaluate the reliability of measurements by two observers (inter-observer variability) and by the same observer one week apart (intra-observer variability). Bland-Altman plots were created in order to visualise the degree of agreement between different inter- and intra-observer measurements, to quantify numerical bias, and to depict potential data outliers.

### Summary of the results

The findings of this study indicate that, in a group of athletes undergoing CMR, HDF parameters showed high reliability. Overall, inter- and intra-observer variability was acceptable (ICC ≥0.85 for all the parameters, except for the intra- and inter-observer variability of Impulse Angle). In terms of bias, the Bland-Altman plots showed minimal bias since the midlines of agreement were close to zero and the upper/lower limits of agreement covered around 95% of inter-observer measurements and 92% of intra-observer measurements (**Figs 3 and 4**).

### Comparison with previous studies

There are only a few studies on the reliability of HDF in humans (**Table 4**).

**Table 4:** Inter- and intra-observer variability of HDF parameters: Comparison with previous studies in humans.

Reference	Imaging	Parameters	Inter-observer ICC (95%C.I.)		Intra-observer ICC (95%C.I.)		Population
Ferrara et al. (Ref. 21)	Echocardiography	Amplitude Heartbeat Longitudinal	0.97 (0.93–0.98)		0.98 (0.95–0.99)		Healthy (n = 30)
Monosilio et al. (Ref. 14)	Echocardiography	A-B L-S	0.85 (0.36–0.96) 0.87 (0.14–0.97)		0.82 (0.22–0.96) 0.76 (0.01–0.94)		Heart failure (n = 10)
Filomena et al. (Ref. 13)	CMR	A-B L-S Impulse Angle	0.98 (0.91–0.99) 0.97 (0.90–0.99) 0.95 (0.83–0.99)		0.98 (0.93–0.99) 0.91 (0.62–0.98) 0.92 (0.67–0.98)		STEMI (n = 10)
Backhaus et al. (Ref. 24)	CMR	Longitudinal force Systolic peak Diastolic deceleration	0.99 (N.A.) 0.98 (N.A.) 0.92 (N.A.)		0.98 (N.A.) 0.96 (N.A.) 0.89 (N.A.)		HFpEF/NCD (n = 5)
Vos et al. (Ref. 23)	CMR	"A"^a^ "B"^a^ "C"^a^ "D"^a^	0.81 (0.52–0.93) 0.88 (0.67–0.96) 0.87 (0.65–0.95) 0.94 (0.84–0.98)		0.75 (0.40–0.91) 0.94 (0.82–0.98) 0.94 (0.82–0.98) 0.99 (0.96–1.00)		Pulmonary hypertension (n = 15)
Lange et al. (Ref. 22)	CMR	Longitudinal force Systolic peak Diastolic deceleration	0.90 (0.1–0.98) 0.87 (0.1–0.97) 0.94 (0.79–0.99)		0.98 (0.91–0.99) 0.97 (0.87–0.99) 0.98 (0.93–1.00)		Healthy (n = 11)
Present study	CMR	A-B L-S Impulse Angle	0.85 (0.67–0.93) 0.86 (0.69–0.94) 0.73 (0.39–0.87)		0.91 (0.78–0.93) 0.93 (0.83–0.97) 0.80 (0.56–0.90)		Athletes (n = 25)

^a^"A": Systolic ejection force; "B": Downward force at systolic-diastolic transition; "C": E-wave decelerative force; "D": A-wave acceleration; HFpEF: Heart failure with preserved ejection fraction; NCD: Non cardiac dyspnea.

Ferrara et al. [21] reported HDF values in healthy individuals using echocardiography. Although several HDF parameters were measured, inter- and intra-observer variability is reported for longitudinal HDF over the whole cardiac cycle. Compared to our results, they found a lower inter- and intra-observer variability (ICC 0.97 vs 0.85 and 0.98 vs 0.91, respectively).

Two studies from the same institution in patients after MI and in patients with chronic HF provide interesting data. Both studies used the same software we have used in our study; however, Filomena et al. [13] analysed CMR while Monosilio et al. [14] analysed echocardiographic images. The former study reported very high ICC (both for inter- and intra-observer variability), while the latter reported values in line with ours. Based on these results, the best imaging modalities in terms of variability is still under debate.

Other recent studies used the same approach as our study in healthy subjects [22] and in patients with pulmonary hypertension [23] or HF with preserved ejection fraction [24]; however, they looked into different parameters, according to the different cardiac phases, while we considered HDF throughout the entire cardiac cycle.

### Potential sources of variability

In addition, we looked into the potential sources of variability according to the different steps of image analysis, which include slice selection, cardiac frame selection, EBD, and VA measurement.

In case of CMR images acquired with perfect planimetry, the observers selected the middle slice from a stack, thus the selection of the slice is highly reproducible. However, in cases when planimetry was not done accurately during scanning, the observers had to search for a compromise when selecting the slice to make it as close as possible to these criteria. On many occasions, the slice is somewhat next to the middle one in the stack. In such a situation, minimal reliability between observers can be observed.

For the cardiac frame selection, a moderate source of variability was found. It is important to note that the measurements on ES and ED frames selected by the observers were done before the observers could refer to the maximal and minimal volume curve presented for the entire heart cycle and finalize their measurements (**Fig 2, Panel B**). Thus, with the aid of the volume curve, it would be possible to achieve a minimal variability.

The GLS shows excellent correlation and statistical significance, which is a consequence of the automatic function of EBD. The observers had to manually adjust the endocardial borders only in 1/25 patients. From these results, we believe that the software is robust enough to be used for follow-up studies.

In terms of the valve measurements, our results indicate a slightly higher inter-observer variability for the AVA compared with MVA (ICC 0.78 vs 0.92). This finding could be explained by the AV annulus having virtual reference points, which are approximately the line connecting the basal points of the AV leaflets. In contrast, MV annulus has definite hinge points that the operator can use as a reference to measure the MV diameter.

Thus, based on our experience, slice selection and VA measurements are the most challenging steps in terms of reliability, while frame selection and endocardial border detection are much less operator-dependent.

Finally, we would like to emphasize the importance of joint training sessions using the software for reducing the degree of variability. Even with experience in cardiac imaging, there should be some agreement on the standardization of measurements.

### Sample size calculation

Sample size calculation is crucial when designing any study, as it dictates the number of subjects needed to detect a desired difference of measurements [25,26]. In the present study, required sample size varied between the different HDF parameters, with the largest required sample size to detect a 10% change for A-B. This value is >3 times higher than the one calculated by Lange et al. [23], which can be explained by the different parameters analysed. In contrast, changes of the directional distribution (angles) of HDF across the entire heart cycle can be detected with a very small sample size, which can be explained by the fact that impulse angle does not take into account the absolute values of the HDF.

### Limitations of the study

Despite the low variability in the observer measurements, there are some aspects that must be considered. The software program only allows the rounding down to one decimal for the diameter of the valves. The measurement of the MV at the annulus and the AV at the aortic annulus may not reflect the actual area through which the blood flows. It is not clear if observers with less experience in cardiac imaging will achieve the same degree of reliability as found in our study. Biological variability cannot be excluded, thus the same results may not apply to patients with distorted left ventricles or heart valve diseases, where endocardial border detection and valve area measurements might be challenging. Finally, training intensity and duration might influence HDF values, however these factors are not relevant for reliability assessment.

## Conclusions

The results of this study show a low inter- and intra-observer variability for the analysis of HDF parameters using CMR images. Image frame selection and VA measurements are the most vulnerable steps in terms of reliability of this method. These findings will likely open new avenues for this method for wide application in clinical settings, in particular for patients undergoing follow-up studies and for early detection of cardiac dysfunction.
